# Optimized Synthetic Flavonols Support Senescence Clearance and Lung Fibrosis Resolution

**DOI:** 10.1021/acsptsci.5c00231

**Published:** 2025-09-04

**Authors:** Jeffrey A. Meridew, John A. Vu, Daniela Chow, Ana Maria Diaz Espinosa, Namita Saraf, Ashley Y. Gao, Jair Machado Espindola-Netto, Sara Dresler, Madison G. Whaley, Kyoung M. Choi, Yong Li, Helene Martini, Eva Carmona Porquera, Patrick A. Link, Thomas M. Kollmeyer, Joao F. Passos, Marissa J. Schafer, Nathan K. LeBrasseur, Daniel J. Tschumperlin, Andrew J. Haak

**Affiliations:** † Department of Physiology and Biomedical Engineering, 6915 Mayo Clinic, Rochester, Minnesota 55902, United States; ‡ College of Medicine and Science, Mayo Clinic, Rochester, Minnesota 55905, United States; § Center for Clinical and Translational Science, Rochester, Minnesota 55902, United States; ∥ Department of Molecular Pharmacology and Experimental Therapeutics, Mayo Clinic, Rochester, Minnesota 55902, United States; ⊥ Robert and Arlene Kogod Center on Aging, Mayo Clinic, Rochester, Minnesota 55905, United States; # Thoracic Diseases Research Unit, Departments of Medicine and Biochemistry, 12270 Mayo Clinic College of Medicine, Rochester, Minnesota 55905, United States

**Keywords:** Lung, Fibrosis, Senescence, Senolytics, Flavonol, Aging

## Abstract

Idiopathic pulmonary fibrosis (IPF) is a progressive and fatal disease with undefined etiology and minimally effective therapies. The greatest risk factor for developing IPF is aging. The central paradigm to developing antifibrotic drugs for the last half century has focused on directly targeting proliferative lung fibroblasts. However, recent high-resolution analyses of IPF patient lungs suggests disease unique populations of resident lung cells are enriched for markers of senescence. Published work by our group and others further supports that senescent cells are key drivers of fibrosis and may provide an opportunity to develop an effective antifibrotic drug. Multiple naturally derived flavonoids can selectively induce apoptosis in senescent cells (senolytic) and improve end points in models of lung fibrosis; however, these natural phytochemicals are not structurally optimized to maximize their translational potential. Inspired by this opportunity we have performed hit-to-lead studies and medicinal chemistry optimization to generate a novel synthetic flavanoid (F-4N) with ∼ 50× greater senolytic potency in vitro- compared to fisetin or quercetin, two naturally derived senolytic flavonols. Furthermore, in bleomycin injury models of lung fibrosis we have shown treatment with F-4N (10 mg/kg-30 mg/kg, daily) promotes reduced senescence burden, resolution of chronic lung fibrosis, and markers of enhanced alveolar epithelial repair.

## Graphical abstract

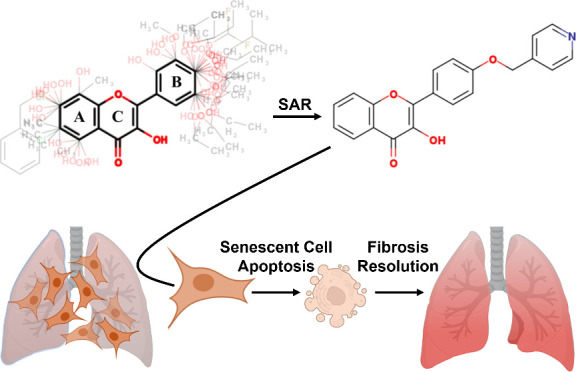

Idiopathic pulmonary fibrosis is a chronic lung disease characterized by progressive interstitial lung fibrosis leading to a rapid decline in pulmonary function and ultimately pulmonary failure within two to five years.^1,2^ The pathogenesis of IPF remains not fully elucidated but has been linked to many senescence-associated processes, such as telomere shortening, DNA damage, and apoptosis inhibition.^1,3^ It is, therefore, unsurprising that advanced age is the primary risk factor for IPF in both males and females,^1^ and, with increases in global life expectancy and improved diagnostic technologies, IPF-related mortality is rising.^4,5^ Currently approved IPF therapeutics nintedanib and pirfenidone have been shown to slow lung function decline in IPF patients but have a limited capacity to improve mortality.^6–9^ The modest benefit that these therapies offer drives efforts to develop more effective and tolerable therapies. Our group and others have previously shown senescent cell populations are major drivers of lung fibrosis and experimental strategies to selectively clear pathological senescence cells are a promising avenue for therapeutic translation.^10–13^

Flavonols are a subclass of natural flavonoid phytochemicals sharing a common chemical scaffold. Along with many other flavonols, quercetin and fisetin have been widely explored for their medicinal properties and potential to treat various diseases.^14–16^ Concerning fibrosis, quercetin and fisetin have shown efficacy in experimental models of multiple fibrotic tissue diseases including lung, liver, kidney, and heart.^17–20^ Recently, flavonols have emerged as some of the first *senolytic* compounds that selectively kill senescent cells while introducing minimal toxicity to healthy proliferative cells.^21^ The discovery of their senolytic effects has sparked renewed interest in flavonols as potential treatments for age-related diseases like IPF,^11,22^ leading to an early stage clinical trial that investigated the safety and efficacy of quercetin (Q) combined with dasatinib (D), another senolytic drug, in IPF patients.^23^ However, consistent with many natural products, quercetin is challenged by its low potency and poor pharmacokinetic characteristics.^24^ To overcome these challenges, we employed a medicinal chemistry hit-to-lead approach with natural and synthetic flavonols to discover a promising lead molecule that is able to exploit the natural senolytic features of flavonols but with improved lead molecule characteristics supported by pharmacokinetic and efficacy studies in multiple rodent models of pulmonary fibrosis.

## Results

### B-Ring, para-Ethoxy Substitution Drives Potency and Selectivity of Synthetic Flavonols

In order to develop and explore the structure–activity relationship (SAR) of flavonol senolytics we cultured human lung fibroblasts to replicative senescence (18–20 passages), confirmed by comparing proliferation, expression of *CDKN1A* and *CDKN2A* and SA-β gal staining to low passage lung fibroblasts, from the same donor (Figure 1A-C). We previously developed a high-throughput assay to quantify expression of cleaved caspase-3 (CCasp3), a hallmark of apoptosis, by high-content immunocytochemistry,^25^ and, here, utilized this platform to compare dose–response effects of natural flavonols quercetin (**Q**) and fisetin (**F**) (Figure 1D) to induce CCasp3 expression in senescent lung fibroblasts vs low passage, proliferating lung fibroblasts. Consistent with published findings, **Q** and **F** displayed senolytic effects only above 30 μM.^10,26–28^

**Figure 1:**
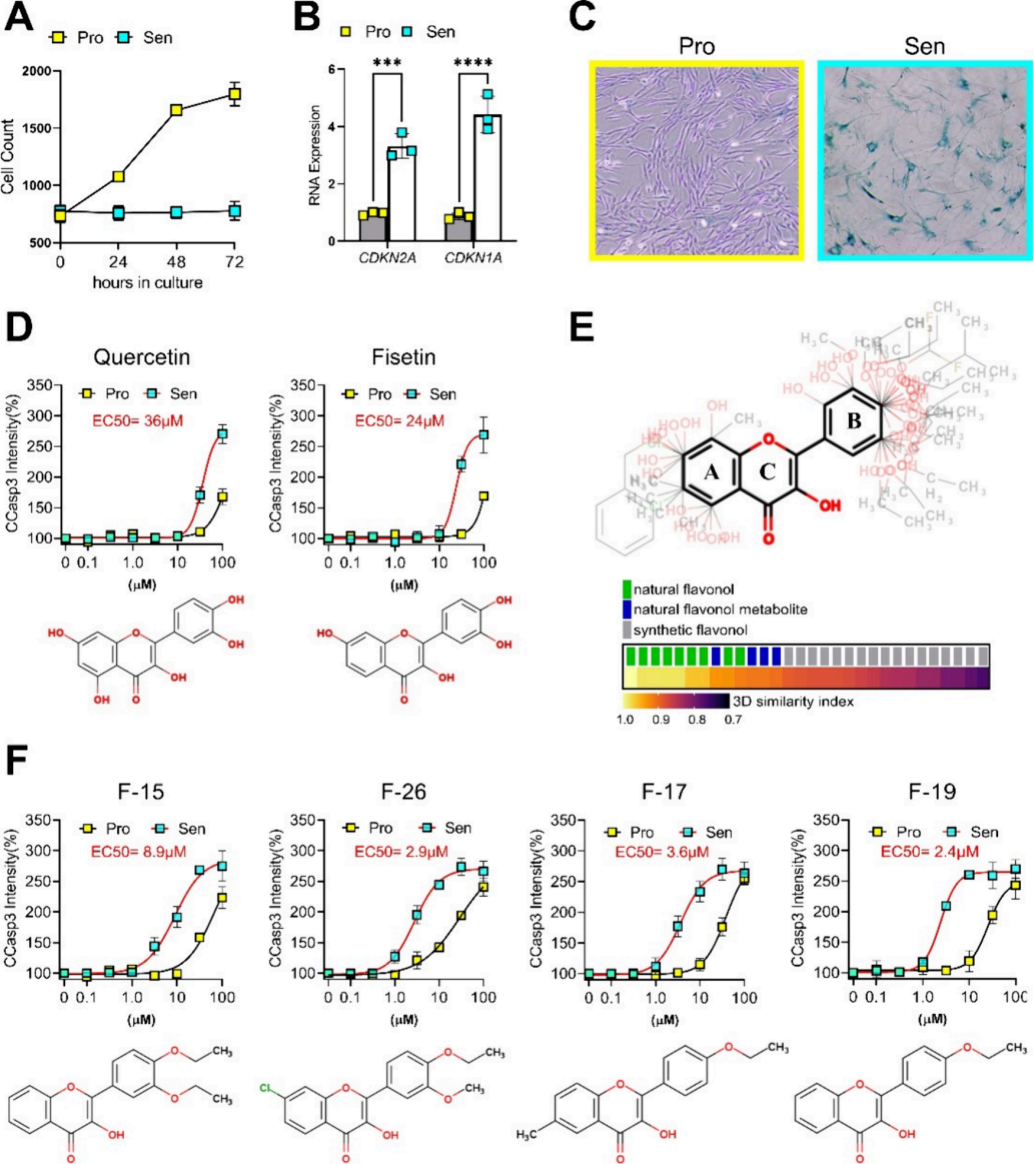
Synthetic flavonols containing a para-ethoxy substitution on the B ring potently and selectively induces apoptosis in senescent lung fibroblasts. Human lung fibroblasts were passaged in culture (*P* = 18–20) until they presented with markers associated with cellular senescence, referred to as “Sen” and compared against the same cell lines at passage 3 “Pro” in assays measuring: **A**. cellular proliferation, **B**. qPCR analysis for transcript expression of cyclin dependent kinase inhibitors- *CDKN2A* and *CDKN1A*, and **C** senescence associated β-galactosidase staining. **D**. Dose–response curves comparing natural product flavonols quercetin and fisetin to induce cleaved caspase-3 staining in Sen vs Pro fibroblasts, measured by immunocytochemistry staining after cells were treated for 72 h with the indicated concentration of flavonol. **E**. Structural diversity of commercially available flavonols. 2-dimensional structural overlay with each flavonol shown at 90% transparency and A, B, and C ring nomenclature identified. Below, a Tanimoto metrics similarity index was calculated, relative to quercetin for each flavonol to highlight the greater chemical diversity opportunity in synthetic flavonols compared to naturally derived. **F**. Senolytic cleaved caspase-3 staining for each of the most potent synthetic flavonols identified in the screen. All data are plotted as the mean ± SEM from three biologically independent experiments. Comparisons made using *t* test, *** *p* < 0.001, **** *p* < 0.0001 vs the indicated group.

One of the limitations with naturally derived flavonols is the narrow chemical diversity presented on their A and B ring substitutions; with only the number and location of hydroxyl groups being the sole driver of differentiation.^29^ We generated a custom diversity library of commercially available flavonols encompassing natural products, natural product metabolites, and synthetic flavonols and generated a 2D structural overlay to visualize consistent and divergent moieties across the molecules. We then calculated Tanimoto similarity indexes for each compound relative to quercetin to visualize the unique diversity provided by synthetic flavonols (Figure 1E). We first triaged the senolytic potential of our flavonol diversity library by measuring viable cell counts in senescent lung fibroblasts after 72 h in culture with 1, 10, and 100 μM of each compound (Supplemental Figure 1). Any compounds that had a clear impact on reducing senescent cells at 10 μM were selected for followup dose–response experiments in senescent and proliferating lung fibroblasts. **F-15**, **F-26**, **F-17**, and **F-19** were found to be the most potent and selective senolytics (Figure 1F), and intriguingly they all contained a para-ethoxy substitution on their B-ring. **F-19** was the most potent and selective senolytic flavonol we identified from commercially available analogs, with an EC50 of 2.4 μM to induce CCasp3 expression in senescent lung fibroblasts, compared to 25 μM in proliferating lung fibroblasts. **F-19** is 10-fold more potent than the natural products **Q** or **F** and had a much larger window of selectivity in this in vitro model.

### F-19 Treatment Reduces Senescence-Associated Markers and Enhances Expression of Mature Alveolar Epithelia Ex Vivo and In Vivo

To further assess the therapeutic potential of F-19, we leveraged ex vivo lung slice cultures, or *precision-cut lung slices* (PCLS), which are a useful model to study the complex multicellular biology of the lung while preserving native tissue architecture.^30,31^ We generated PCLS from aged mouse lungs ± bleomycin-induced fibrosis and treated with **F-19** or the clinically approved antifibrotic, nintedanib, ex vivo for 72 h prior to collecting RNA from the lung slices for qPCR analysis. Compared to vehicle-treated, both compounds reduced expression of *Col1a1*, one of the two genes encoding type I collagen, the most abundant extracellular matrix (ECM) protein in the lung (Figure 2A). Compared to vehicle- and nintedanib-treated, **F-19**-treated PCLS had significantly reduced expression of senescence-associated markers *Cdkn2a*, *Cdkn1a*, and *Il6*. **F-19** also uniquely increased expression of *Sftpc* and *Hopx*, markers of mature alveolar epithelium, suggesting the possibility that removal of senescent cells from fibrotic lung could both stop interstitial fibrosis and stimulate or facilitate alveolar regeneration, a finding consistent with previously reported effects of senolytic targeting in fibrotic lungs ex vivo^32^ (Figure 2A).

**Figure 2:**
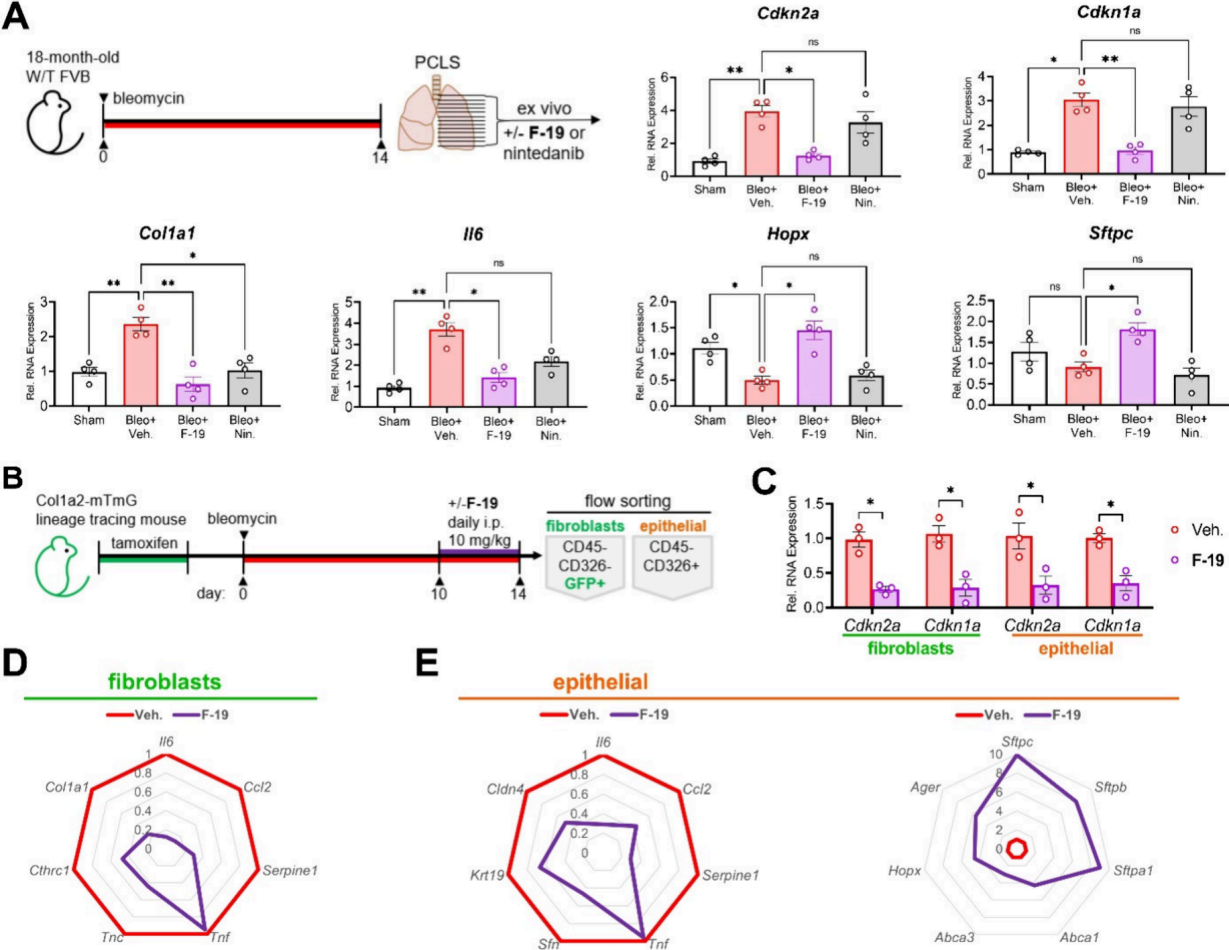
Synthetic flavonol F-19 supports clearance of senescence associated markers ex vivo and in vivo. **A**. Aged (18-month-old) mice were administered intratracheal “sham” or bleomycin (Bleo) and 14 days after exposure the left lobe was harvested to generate 300 μm precision-cut lung slices that were cultured for 72 h with F-19 (10 μM) or Nintedanib (1.0 μM) ex vivo, prior to homogenization, RNA isolation and qPCR analysis. *N* = 4 sham or bleomycin mice. Comparisons made using ANOVA, * *p* < 0.05, ** *p* < 0.01 vs the indicated group. **B**. Young (2-month-old) Col1a2-mTmG, fibroblast lineage tracing mice were prelabeled with tamoxifen, 14 days prior to intratracheal bleomycin administration. Starting on day 10 after bleo injury, one group of mice was treated with F-19 (daily, 10 mg/kg, i.p.) for 4 days. **C**. On day 14 post Bleo the lungs were harvested for flow sorting (FACS) to collect populations of fibroblasts and epithelial cells for RNA isolation and qPCR analysis of *Cdkn2a* and *Cdkn1a*. All data are plotted as the mean ± the SEM from three mice. Comparisons made using *t* test, * *p* < 0.05 vs the indicated group. **D** and **E**. Radar plots of additional transcripts analyzed from the fibroblasts and epithelial cell populations including senescence associated inflammatory cytokines, profibrotic ECM genes, markers of mature alveolar epithelial cells, and markers of aberrant epithelial cells associated with IPF. All data are plotted as the mean, F-19 treated mice (purple), relative to the mean, vehicle treated mice (red, set to 1). Data are also provided as individual plots in Supplemental Figure 2.

Pathologically unique, mesenchymal and epithelial cells expressing markers of senescence have been identified in experimental models of lung fibrosis and IPF patient lungs.^33–38^ To investigate the specific effect of **F-19** on fibroblasts and epithelial cells we generated a mesenchymal lineage tracing mouse by breeding Col1a2-CreERT(2) mice (Jackson Laboratory) with Rosa26-mTmG mice. To permanently lineage-label the Col1a2+ population in these mice, each mouse was administered tamoxifen (75 mg/kg) for 5 days, starting 15 days prior to lung injury^39^ (Figure 2B). On day 10 after bleomycin injury, we began treatment with 10 mg/kg i.p. **F-19** daily injections for 4 days. Lungs were then harvested, and flow-sorted to collect fibroblasts and epithelial cells. RNA from each population of cells was collected and analyzed by qPCR. Compared to vehicle-treated mice, the **F-19**-treated mice had significantly reduced expression of senescence-associated genes *Cdkn2a* (p16) and *Cdkn1a* (p21) in both fibroblasts and epithelial cells (Figure 2C). We next expanded transcript analysis to include ECM-associated genes enriched in fibrotic lung fibroblasts:^38,40^ *Col1a1*, *Tnc*, and *Cthrc1*, senescence-associated soluble factors: *Il6, Ccl2*, and *Serpine1*, and markers of the aberrant basaloid/intermediate epithelial population unique to fibrotic lungs:^33,41^ *Cldn4, Krt19*, and *Snf*. In all cases, **F-19** treatment normalized expression of these pathological markers of lung fibrosis (Figure 2D-E, individual gene plots are also provided in Supplemental Figure 2). Consistent with our ex vivo findings **F-19** treatment elevated expression of alveolar epithelial ATII markers: *Sftpc*, *Sftpb, Sftpa1*; ATII/ATI markers: *Abca1, Abca3*; and ATI markers: *Hopx* and *Ager*, compared to vehicle-treated mice. Interestingly, **F-19** did not alter expression of TNF-α in either cellular compartment, which is potentially advantageous, as TNF-α has previously been shown to be beneficial in lung fibrosis resolution,^42^ further supporting the potential of optimized senolytic flavonols and highlighting the nuanced nature of this approach as not simply an “anti-inflammatory” effect.

### Novel Flavonol F-4N Potently and Selectively Induces Apoptosis in Senescent Lung Fibroblasts

In an effort to improve solubility, potency, and selectivity we designed and synthesized a short series of additional para-ethoxy substituted analogs of **F-19** and measured their senolytic potential using the CCasp3 assay (Table 1). To benchmark our flavonols against clinically developed and approved therapies also known to selectively induce apoptosis in senescent cells,^43,44^ we also tested the potency and selectivity of Bcl-2 family inhibitors ABT-263 (navitoclax), ABT-199 (venetoclax), and ABT-737 in the CCasp3 assay comparing senescent and proliferating lung fibroblasts (Table 1). Concerning our medicinal chemistry findings within the flavonols, we first increased the alkyl chain of **F-19** from ethyl to propyl which was largely tolerated and then substituted a phenyl group which displayed a modest improvement in potency and selectivity (**F-20**, Table 1), however this was accompanied by a predictable loss in solubility which we recovered with a pyridine. Walking the nitrogen around the ring turned out to be critical, as position 4 (**F-4N**) had a vastly superior potency (EC50 = 900 nM) and produced an excellent senolytic window (61.1-fold selective) (Table 1, Figure 3A-B). We confirmed caspase 3/7 activity using a luminescent substrate (Figure 3C) and validated F-4N led to selective apoptosis in senescent lung fibroblasts without an observable impact on proliferating lung fibroblasts (Figure 3D). We also confirmed F-4N could target senescent fibroblasts generated by multiple methods including radiation and DNA damaging chemotherapeutics (Supplemental Figure 3). We set out to generate an optimized senolytic flavonol with lead molecule characteristics, one of the major limitations of the natural flavonols is the rapid metabolism.^45^ We hypothesized that removing the catechol feature on the “B” ring would promote stability of F-4N compared to quercetin as the catechol is known to be a substrate for O-methyl-transferases and oxidation.^46,47^ In support of this, we observed the half-life of quercetin to be ∼ 30 min and F-4N to be >120 min in plasma from both mouse and human (Supplemental Figure 4). The pharmacodynamic and physicochemical improvements of **F-4N** over **Q** and **F-19** met our criteria of a lead molecule to move forward into ex vivo and longer treatment in vivo studies to look at end points in chronic models of lung fibrosis. To compare senolytic and pro-reparative effects of these compounds in fibrotic lung tissue, we again performed PCLS experiments generated from 18-month-old mice ± bleomycin injury and compared **F-4N** to Bcl-2 inhibitors. Interestingly, we found either class of compounds supported reductions in senescence associated markers but only **F-4N** stimulated expression of markers indicative of alveolar regeneration, suggesting beneficial or detrimental effects of these molecules may extend beyond senolysis and are likely scaffold or mechanistically unique (Supplemental Figure 5).

**Table 1:**
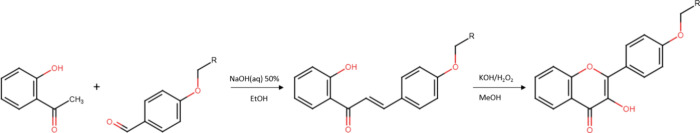
Synthesis and Senolytic Potency/Selectivity of Novel Flavonols

Compound	R	cLogP	Senescent fibroblast apoptosis EC50 (μM)	Proliferating fibroblast apoptosis EC50 (μM)	Senolytic window (EC50^Pro^/EC50^sen^)
F-19	CH_2_	2.92	2.4	24.5	10.2
F-19(Me)	CH_2_CH_3_	3.44	3.4	19.5	5.7
F-20	phenyl	4.29	1.5	27.6	18.4
F-2N	2-pyridine	3.15	21.8	29.8	1.4
F-3N	3-pyridine	3.07	11.2	14.9	1.3
F-4N	4-pyridine	3.07	0.8	48.9	61.1
ABT-737	-	-	0.7	9.7	13.0
ABT-199	-	-	3.2	76.5	23.9
ABT-263	-	-	1.9	14.9	7.8

**Figure 3:**
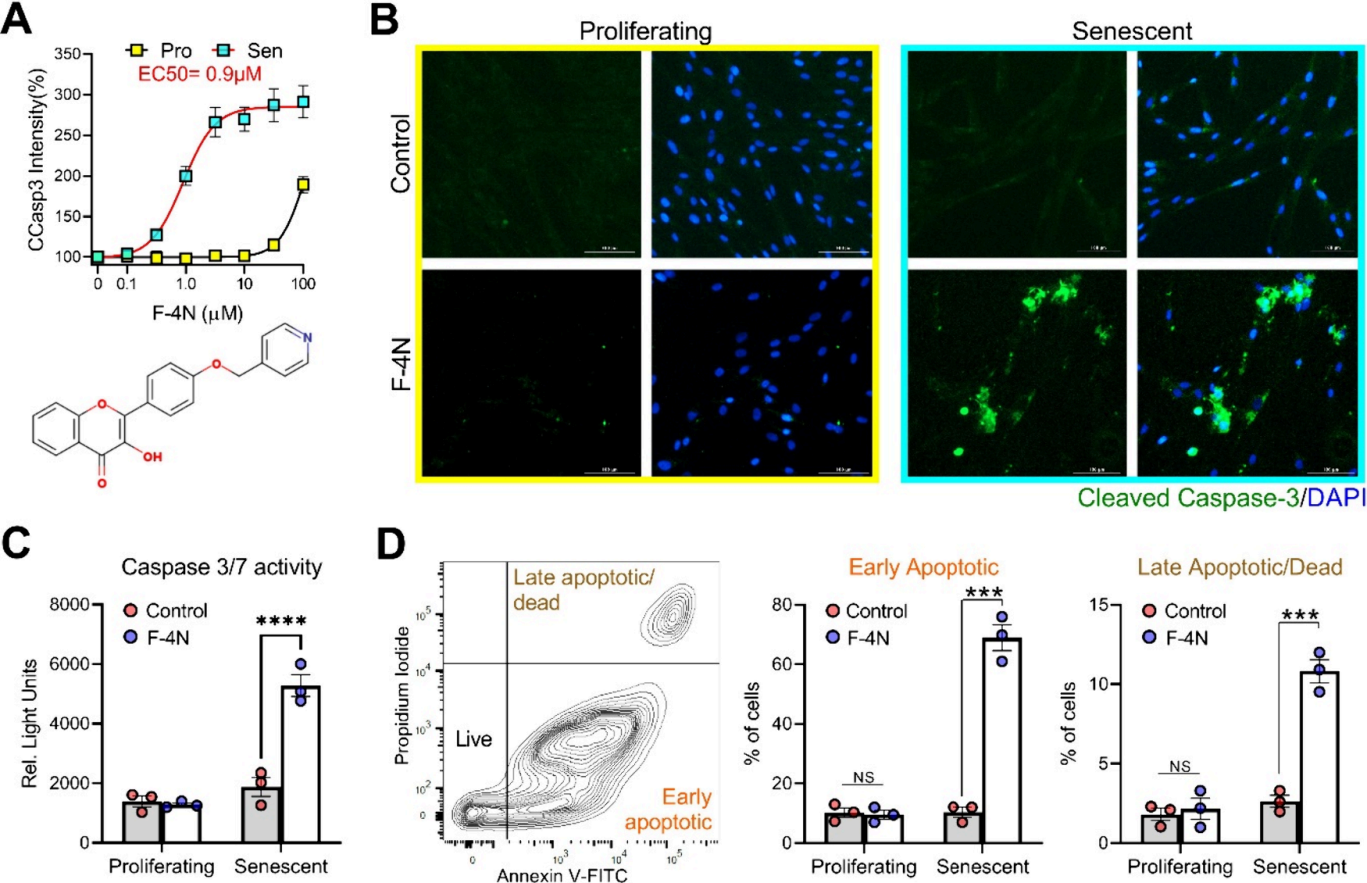
Discovery of F-4N, a potent and highly selective senolytic flavonol. **A**. Dose–response curves comparing F-4N capacity to induce cleaved caspase-3 staining in replicative Sen vs Pro fibroblasts, measured by immunocytochemistry after cells were treated for 72 h with the indicated concentration. **B**. Representative images of proliferating or replicative senescent lung fibroblasts treated for 72 h ±F-4N (3.0 μM) then stained for Cleaved-Caspase-3 and DAPI. Scale bar represents 100 μm. **C**. Caspase 3/7 activity measured by a luminescent substrate after 72 h ± F-4N (3.0 μM). **D**. Flow cytometry-based analysis of early and late apoptosis measured by Annexin V and Propidium Iodide staining. Shown is a representative contour plot from an experiment with senescent fibroblasts treated with F-4N for 72 h and quantification of early and late apoptosis ±F-4N (3.0 μM) in proliferating and senescent cells. *N* = 3 independent experiments (***p < 0.001, ****p < 0.0001 vs the indicated group).

### F-4N Resolves Lung Fibrosis and Survival In Vivo

To assess the efficacy of F-4N in robust models of rodent lung fibrosis, we first performed a study in aged mice, delivering bleomycin one-time to produce chronic, nonresolving fibrosis.^48–50^ On day 14 after bleomycin injury, after fibrosis is established, we began treatment with 10 mg/kg **F-4N** or vehicle by i.p. daily injections for 14 days. At the completion of the study, we observed an improvement in mouse survival in the group treated with F-4N, compared to vehicle-treated mice (Figure 4A). F-4N also elicited marked improvements in overall lung architecture and collagen staining, and a nearly complete resolution of lung collagen content, compared to vehicle-treated mice, as measured by lung hydroxyproline content (Figure 4B). In vivo measurements of whole lung transcript levels of pathological markers associated with ECM production and senescence-associated genes were also consistent with our ex vivo studies. Compared to vehicle-treated mice, F-4N-treated mice had significantly lower expression of ECM and senescence-associated transcripts, but significantly elevated markers associated with alveolar regeneration and repair (Figure 4C). We also measured plasma levels of IL-6, a senescence-associated biomarker and observed a nearly complete normalization (Figure 4D). A second model of chronic lung fibrosis in mice is repeated delivery of bleomycin every 2 weeks for 6 weeks in young mice.^51^ We performed an analogous dosing/treatment study with **F-4N** using this repeated bleomycin model and observed features consistent with resolution in the F-4N-treated mice compared to vehicle-treated mice: improvements in body mass, lung architecture, collagen content, and whole lung gene expression changes (Figure 5). In particular, we measured expression of mature alveolar epithelial markers *Hopx*, *Ager*, *Sftpa1*, and *Sftpc*, again observed F-4N enhanced expression while repressing expression of markers of the aberrant basaloid/intermediate epithelial population unique to fibrotic lungs:^33,41^ *Snf*, *Cldn4*, *Krt19*, and *Mmp7* (a putative biomarker that is predictive of lung fibrosis progression and mortality^52–54^ ) (Figure 5C). We next investigated the potential of F-4N to be dosed orally for future studies. To set an initial benchmark we compared the mouse liver microsomal stability of quercetin to our lead molecule and found the half-live of F-4N to be >3-fold higher than quercetin (Supplemental Figure 6). Finally, we performed an in vivo study to define plasma levels following oral delivery of F-4N after 7 days of treatment (10, 30, and 100 mg/kg), 2 and 8 h after the final dose in mice with bleomycin induced lung fibrosis (Figure 6A-B). From this same study we sought to define the relationship between plasma levels of F-4N and efficacy at reducing markers of the SASP, so we isolated whole lung RNA and measured transcript expression of 84 different cytokines and chemokines comparing the vehicle treated mice to the 30 mg/kg treated mice (Figure 6C). 30 mg/kg was selected for transcript studies based on the observed improvement in bodyweight and mean plasma F-4N levels at 2 h measuring 561.4 ng/mL (1.62 μM) which is approaching 2-fold greater than the defined in vitro IC50 (0.9 μM). We selected the top six genes (*Infa2*, *Il6*, *Ccl19*, *Cxcl13*, *Tgfb2*, *Ltb*) reduced by F-4N for comprehensive comparisons across all study groups to confirm that 30 mg/kg is the minimum effective oral dose to impact expression in this model (Figure 6D).

**Figure 4:**
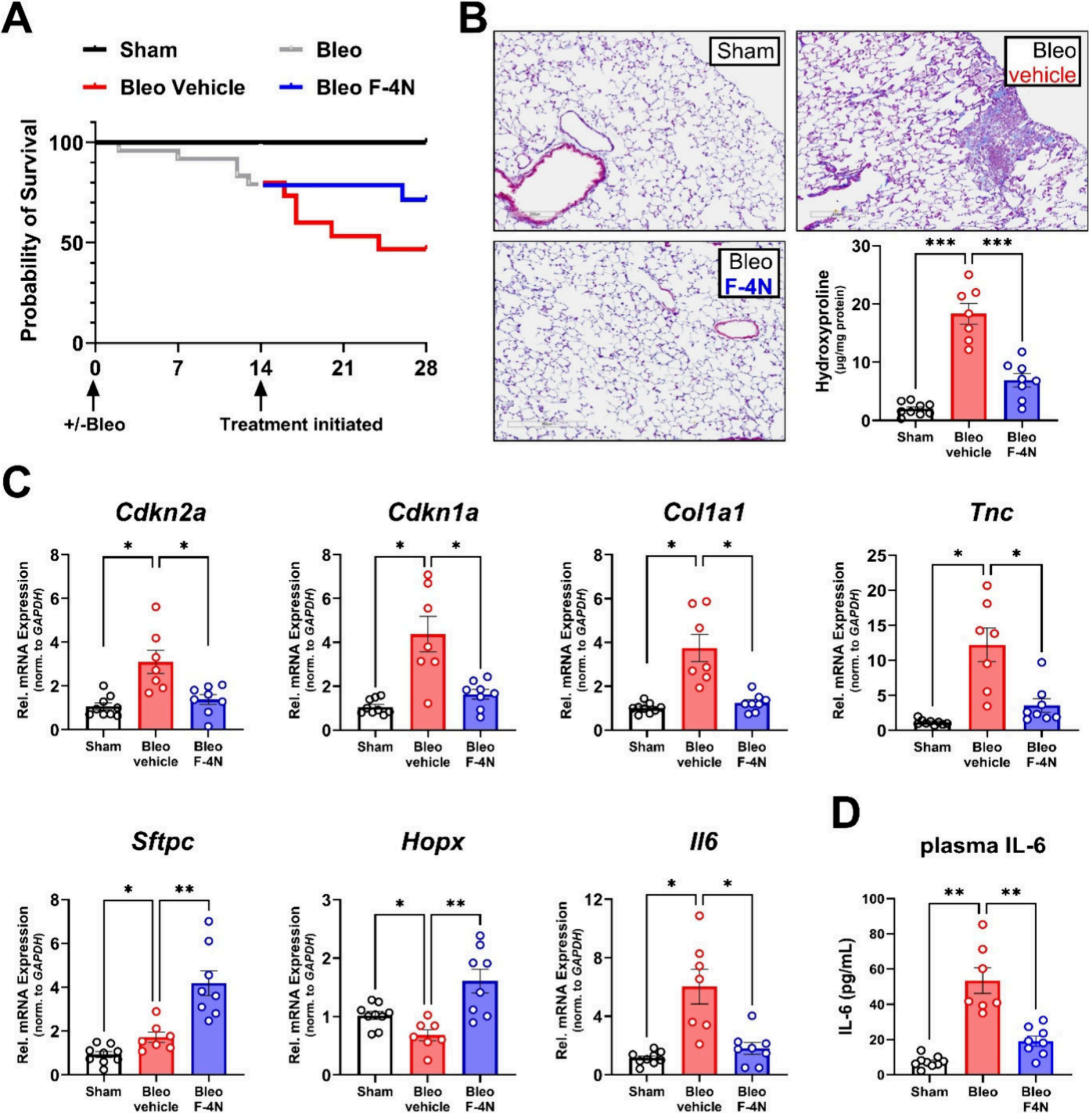
F-4N efficacy in a bleomycin-induced model of lung fibrosis in aged mice. **A**. Survival and study design. Bleomycin was administered one time intratracheally, on day 14 mice began receiving F-4N (10 mg/kg, daily i.p.) or vehicle and lungs were collected on day 28. **B**. Representative Masson’s trichrome stained histological sections and hydroxyproline content measured from whole left lung. Scale bar = 100 μm **C**. RNA Expression of senescence associated markers, ECM genes, and mature alveolar epithelial markers from whole lung at the completion of the study. **D**. Plasma IL-6 levels. *N* = 7–9 mice/group, male and female mice (*p < 0.05, **p < 0.01, ***p < 0.001 vs the indicated group).

**Figure 5:**
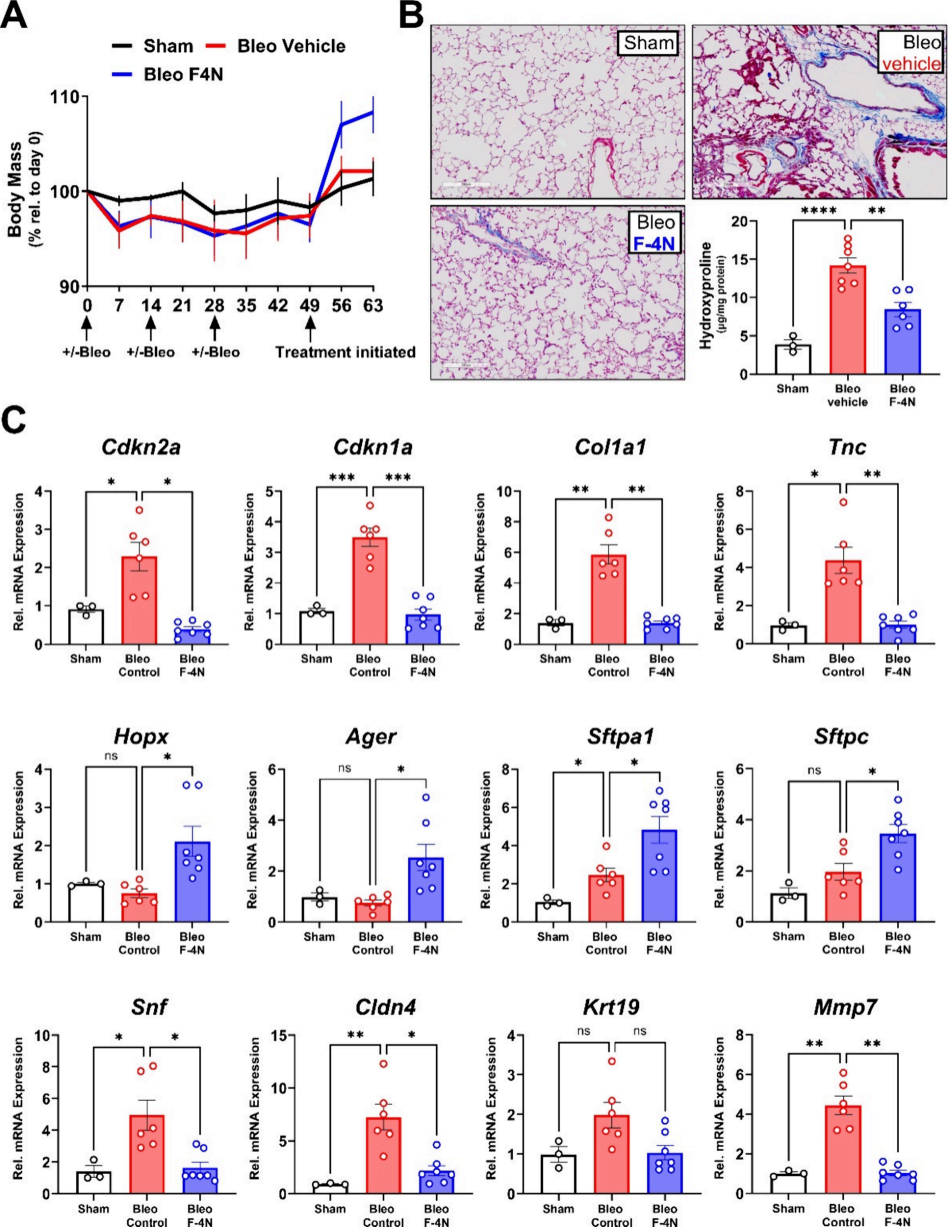
F-4N efficacy in a repeated bleomycin-induced model of lung fibrosis. **A**. Body mass and study design. Bleomycin was administered three times intratracheally, starting on day 0 and repeated on days 14 and 28. Three weeks after the final bleomycin administration, mice began receiving F-4N (10 mg/kg, daily i.p.) or vehicle for 2 weeks prior to lung collection. **B**. Representative Masson’s trichrome stained histological sections and hydroxyproline content measured from whole left lung. Scale bar = 100 μm. **C**. RNA Expression of senescence associated markers, ECM genes, mature alveolar epithelial markers, and aberrant epithelial cells markers from whole lung at the completion of the study. *N* = 3–7 mice/group, male and female mice (*p < 0.05, **p < 0.01, ***p < 0.001, ****p < 0.0001 vs the indicated group).

**Figure 6:**
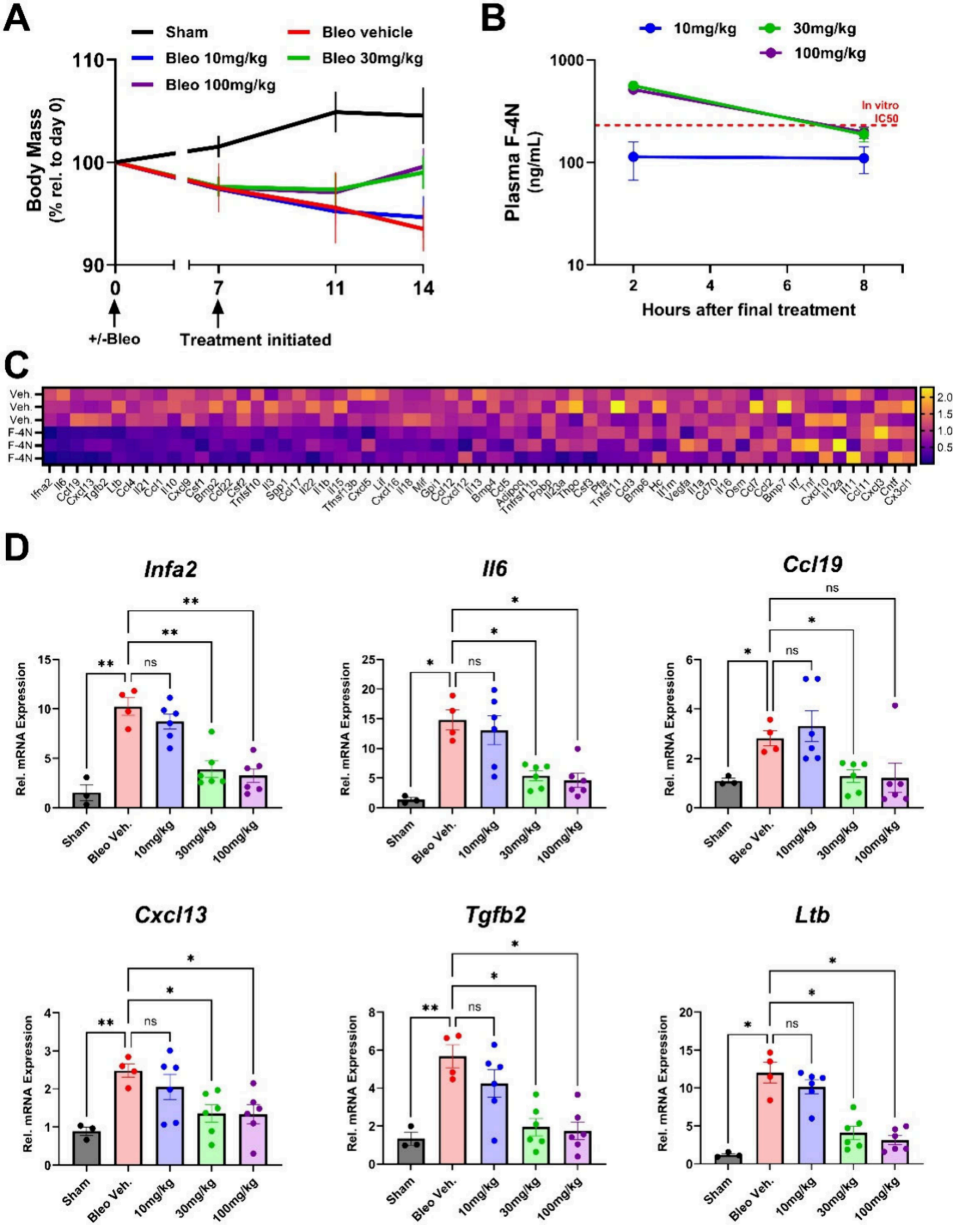
Oral dose-finding study with F-4N. **A**. Body mass and study design. Bleomycin was administered one time intratracheally, on day 0. One week after bleomycin administration, mice began receiving F-4N (10, 30, or 100 mg/kg, daily p.o.) or vehicle for 1 week prior to plasma and lung collection. **B**. Two and 8 h after the final dose, on day 14, plasma was collected and analyzed by LC-MS for unbound (free) F-4N. Three mice/group/time point. Analysis performed under contract by Cyprotex. **C**. Whole lung RNA from the three vehicle and three 30 mg/kg treated mice was collected and transcript levels of 84 cytokines and chemokines was quantified and plotted as a heatmap. Each gene was normalized to the same Veh. treated mouse and expressed as relative fold-change. **D**. The top six genes impacted by F-4N were analyzed by qPCR across all study groups. *N* = 3–6 mice/group, male and female mice (*p < 0.05, **p < 0.01, vs the indicated group).

## Discussion

Senolytic compounds were first identified in 2015 by targeting members of prosurvival gene networks that were sensitive to RNAi in senescent cells but not in proliferating or quiescent cells.^21^ A plant derived flavonoid, quercetin, was one of the founding members of this new class of molecules with promising therapeutic applications. The pervasive appearance of senescent cells in essentially all age-related diseases has spawned a series of early phase clinical trials to assess the safety and efficacy of senotherapeutics, including quercetin and fisetin, comprehensively catalogued in multiple reviews.^51,55,56^ The pharmacodynamic limitations (EC50 > 30 μM) and pharmacokinetic limitations (poor bioavailability, rapid metabolism) motivated our effort here to explore quercetin as a *hit* rather than a *clinical candidate*. We employed traditional medicinal chemistry techniques to identify the key features of the compound necessary for its activity, resulting in the discovery of several synthetic analogs with enhanced *lead-like* properties. An additional motivation for this work is the exclusion of patients with pulmonary arterial hypertension (PAH) from senolytic clinical studies involving dasatinib + quercetin. This is due to studies demonstrating an increase in development of PAH in patients that were given dasatinib to treat chronic myeloid leukemia, and evidence that dasatinib causes pulmonary endothelial toxicity.^57–59^ Pulmonary hypertension is more common in patients with advanced IPF and, in some studies, more than half of IPF patients also have PAH.^60^ In addition to the risk of developing pulmonary hypertension, there is a growing body of evidence to suggest vascular rarefaction and loss of microvascular endothelial identify plays a critical role in the pathology of IPF,^48,61–63^ highlighting a limitation of the senolytic cocktail D+Q for the treatment of lung fibrosis.

Consistent with what we observed, it was previously found that treating fibrotic alveolar epithelial cells and ex vivo lung tissue cultures with D+Q reduces both senescence-associated secretory phenotype (SASP) and ECM markers while increasing markers of alveolar repair.^32^ This potential dual action of eliminating senescent cells while stimulating regeneration from healthy stem cells raises the question as to the mechanism by which flavonols could support regeneration. It has been suggested that the SASP induces dysfunction in alveolar epithelial stem cells^64,65^ and stimulates a profibrotic state in lung fibroblasts.^64,66^ It is therefore possible that removal of a profibrotic senescent epithelial cell population leads to a decline in chemical stimuli that are inhibiting alveolar regeneration. Another possible explanation for this phenomenon may be that senescent cell clearance exposes epithelial niches that permit or initiate a regenerative program in nearby alveolar stem cells and allow for repair. It is also possible that the regenerative capacity of flavonols is a finding independent of its role as a senolytic compound. Other compounds, such as Bcl-2 inhibitors, are capable of depleting senescence markers but have not been shown to elevate markers of mature alveolar epithelial cells, suggestive of alveolar regeneration, as we have shown here. These questions will be a major focus of our future work. Importantly, a limitation of the studies presented here, we do not compare the in vivo efficacy of F-4N to other established senolytic agents to benchmark senescence clearance and antifibrotic impact.

One of the main chemical biology findings of our screening and hit-to-lead campaign is the identification of the para-ethoxy substitution on the flavonol B-ring driving senolytic potency and selectivity. This feature is best highlighted in **F-19**, one of the most potent flavonols we identified, compared to **F-11**, a compound without any observable senolytic activity (Supplemental Figure 1), despite these molecules only differing by one carbon: F-19 (para-ethoxy) and F-11 (para-methoxy). We acknowledge this is an exceptionally dynamic structure–activity relationship and propose target selectivity as one potential mechanism. Quercetin, like many naturally derived compounds, produces effects in biological assays through an undefined mechanism of action and due to its apparent low potency potentially binds with low affinity to multiple targets.^67–69^ Therefore, we reason the para-ethoxy’s impact on cellular potency is driven by enhancing target selectivity, rather than target affinity, although they are not mutually exclusive. Based on previous work suggesting quercetin inhibits a broad number of kinases,^68,70^ our future work will focus on exploring if F-4N inhibits one of more kinases that drive the senolytic activity observed here. Narrowing down the molecular target will support both the mechanism of action studies, and further optimization toward a clinical candidate. Interestingly, the B-ring is also the major source of antioxidant potent of natural flavonols quercetin and fisetin due to the catechol group’s electron delocalization capacity to neutralize free radicals.^71^ By substituting this for a redox-inert feature while retaining and enhancing potency, we support our hypothesis that these molecules are working through a specific target rather than as antioxidants.

In this work, we have focused on identifying synthetic flavonols with potent senolytic activity in primary human lung fibroblasts and ex vivo and in vivo rodent models of pulmonary fibrosis. We have not yet explored the multitude of disease models where senolytic compounds have shown efficacy.^72–75^ Further advances in uncovering the mechanism of action of senolytic flavonols may offer a path for translation and identifying which pathologies are most likely to benefit from flavonol senolytics. The molecules identified here can serve as molecular tools to study the role of senescence in biology, chemical probes for future target ID efforts, as well as the foundational scaffolds of future clinical candidates.

## Methods

### Sex as a Biological Variable

Our study examined male and female animals, and similar findings are reported for both sexes.

### Cell Culture

Primary human lung fibroblasts (NHLF) were purchased from ScienCell and cultured in DMEM/F12 (Gibco) containing 10% fetal bovine serum (Invitrogen) and Antibiotic-Antimycotic (Gibco).

### Senescence Induction In Vitro

Senescence was induced experimentally by passaging the cells until replication exhaustion, typically passage 18–22, or treating cells with 20 μM etoposide or 10 μM bleomycin for 48 h, followed by 10 days in normal culture media as we have previously performed.^76,77^ Radiation induced senescence was triggered by exposing cells to 20 Gy of X-ray irradiation and culturing for 10- and 12-days postirradiation with media refreshed every 48–72 h, as performed previously.^78^

### Cleaved Caspase-3 Staining and Analysis and Caspase 3/7 Activity Assay

Experiments were performed as previously described.^25^ Briefly, proliferating or senescent cells were added to 96-well plates and allowed to attach. After 6 h, media was changed to EMEM containing 0% FBS and the indicated concentration of compound was added. After 72 h, the cells were fixed with 4% formalin, permeabilized with 0.25% triton X-100, blocked with 1% BSA, and immunostained with an antibody for cleaved caspase-3 (Cell Signaling, 9661), an Alexa Flour-488 secondary antirabbit antibody (Thermo Fisher, A-11008) was incubated along with DAPI. Images were collected using a Cytation5 automated microscope (Biotek), using a 4X objective and cleaved caspase-3 intensity was quantified using onboard software (Gen5). Alternatively, caspase 3/7 activity was measured using a Caspase-Glo 3/7 assay from Promega following the manufacturer’s suggested protocol after the 48-h incubation with compounds. Luminescence was measured using a Promega GloMax plate reader.

### RNA Isolation and qPCR Analysis

Cells or lung tissue homogenates were treated as indicated in each experiment before RNA isolation was performed using the RNeasy Plus Mini Kit (Qiagen), as per the manufacturer’s protocol. Isolated RNA was used to synthesize cDNA using SuperScript VILO (Invitrogen Life Technologies). Fast Start Essential DNA Green Master (Roche) was used to perform quantitative PCR analysis using a LightCycler 96 (Roche) instrument. ΔΔC_t_ calculation was used relative to glyceraldehyde-3-phosphate dehydrogenase (GAPDH) to determine the fold change in gene expression. For the oral dose-finding study (Figure 6) RT^2^ Profiler PCR Array Mouse Cytokines & Chemokines from Qiagen were used according to manufacturer suggestion. A list of primers and their sequences is provided in the Supporting Information document.

### SA-β-Gal Staining

Growth media was removed from the cells before the plate was rinsed with PBS. One mL of the Fixative solution from the Senescence Beta-Galactosidase Staining Kit (Cell Signaling) was added to each well and incubated for 15 min. The plates were rinsed twice with PBS before 1 mL of Beta-Galactosidase Staining Solution (Cell Signaling, 9860) was added to each well. The plate was then incubated at room temperature overnight. The cells were then observed under a microscope and SA-β-Gal staining was detected at 20X objective to identify blue precipitants using an inverted bright field microscope.

### Mice

FVB, mTmG (strain 007576), and Col1a2-CreER (strain 029567) were purchased from Jackson Laboratories to track fibroblast-specific lineage. All animal experiments and procedures were approved by Mayo Clinic’s Institutional Animal Care Committee (IACUC) and performed in accordance with the National Institutes of Health guidelines.

### Bleomycin Lung Fibrosis Model

Tamoxifen (75 mg/kg) was injected intraperitoneally (i.p.) into 10-week-old male and female Col1a2-Cre; mTmG mice for 5 consecutive days, starting 14 days prior to bleomycin administration. Three different bleomycin models were used in these studies: single administration in young mice, single administration in aged mice, and repeated (3X) administration in young mice. Mice were given bleomycin intratracheally (i.t.) 0.6 or 1.0 U/kg (Novaplus) or saline control while under isoflurane anesthesia. Bleomycin was administered once to the Col1a2-cre;mTmG mice at 2 months of age (young) and FVB mice at 15–18 months of age (aged). In the final study, 2-month-old FVB mice received three administrations of bleomycin, every 2 weeks. The Col1a2-Cre;mTmG mice were treated from day 10–14 after bleomycin with i.p. F-19 (10 mg/kg) and harvested at day 14 for flow analysis. In both the aged and repeated models, mice received i.p. F-4N (10 mg/kg) daily from days 14–28 post final bleomycin administration. Mice were sacrificed and samples were collected at day 28.

### Fluorescence-Activated Cell Sorting

Mouse lung epithelial and fibroblast populations were sorted and collected as we have previously performed.^10,48,79^ Mouse primary lung cell suspensions were generated by a combination of mechanical disruption via razor blade and enzymatic digestion in DMEM (Gibco) with Liberase TM (10 μg/mL, Sigma) and DNase I (10 U/mL, Sigma) for 35 min at 37 °C and passed through a 40 μm strainer (Fisher Scientific). Digestion was inactivated by DMEM with 10% FBS and pelleted at 400 × g for 5 min. Samples underwent 90 s of red blood cell lysis (Biolegend) which was inactivated using autoMACS rinsing solution (Miltenyi) and passed through a 30 μm mesh (Sysmex America). Cells were spun down, resuspended, and stained with CD326:APC, CD45:PerCP-Cy5.5, CD31 (Biolegend) and DAPI (Thermo) for 30 min on ice. The cells were sorted using a BD Aria II cell sorter.

### Precision-Cut Lung Slice Model

Mice were anesthetized with an i.p. injection of ketamine/xylazine (150 mg/kg/10 mg/kg). Mice were perfused with PBS with 2 mM EDTA, a tracheotomy was performed, and the lungs were insufflated with 10% porcine gelatin (Sigma) in Hanks’ balanced salt solution (HBSS). Trachea was ligated and lungs removed from the thoracic cavity and placed in cold HBSS (Gibco). Once gelatin solidified, the left lobe was removed and sectioned into 300 μm slices. Slices were cultured for 3 days in 50% normal fibroblast culture media described above and 50% alveolar epithelial media from Sciencell containing the manufacturer’s suggested proprietary growth supplements. We found this media combination to be the most effective at supporting markers of activated fibroblasts and maintaining alveolar epithelial markers ex vivo (unpublished data). 1 μM treatment with nintedanib was selected based on previously published reports demonstrating this to be the approximate peak plasma concentration achieved with 100 mg/kg dosing for nintedanib in mouse studies.^80^

### Flavonol Synthesis

Equimolar amounts (2–8 mmol) of the hydroxyacetophenone were combined with the aldehyde in 10–50 mL of EtOH. 5–12 mL of 50% NaOH­(aq) was added to the reaction and allowed to proceed for 6–48 h at room temperature while monitoring by thin layer chromatography (TLC). After aldehyde depletion, the resulting calchone was precipitated by 10% HCl­(aq). Calchone was isolated, dried, weighed, and dissolved (1–4 mmol) in 8–30 mL of MeOH containing 1 M KOH. 5–15 mL of H2O2 was added to the reaction and allowed to proceed for 1–6 h at room temperature while monitoring by TLC. The final product was precipitated by 10% HCl­(aq) and purified by recrystallization. Structure and purity (>95%) were confirmed by NMR spectra using a Bruker-Spectrospin Ultrashield 500 NMR running TopSpin 3.6.5 software. Spectra in DMSO-d6 were recorded for 1H (500 or 600 MHz) and 13C (126 or 150 MHz) at 25 °C. Residual solvent protons (δ 2.50 ppm) and solvent carbon signal (δ 39.52 ppm) were used as references for 1H and 13C spectra, respectively. ^1^H/^13^C NMR peak descriptions and spectra are provided in the Supporting Information. Purity was also confirmed to be >95% by LC-MS independently, by Cyprotex in preparation for the contracted in vitro and in vivo PK analysis described below.

### In Vitro and In Vivo PK Studies

F-4N and Quercetin mouse and human plasma and mouse microsomal stability studies were performed under contract by Cyprotex. The test compounds (1 μM) are incubated in plasma or microsomes for the indicated times before the reaction is terminated by acetonitrile. Following centrifugation, the disappearance of parent molecule is measured by LC-MS/MS. The half-life and the percentage of test compound remaining at the individual time points relative to the 0 min sample are plotted. A control compound known to be metabolized quickly by plasma esterases, and a control compound known to be stable, are also incubated alongside each batch of test compounds for plasma studies, and a control compound known to be metabolized by liver microsomes is ran alongside each batch of tech compounds for microsomal stability studies. The plasma analysis was also performed by Cyprotex following their traditional assay development workflow using plasma derived from littermate control mice treated with vehicle alone as blank matrix.

## Supplementary Material



## Notes

Three-point dose–response experiments for the original 30 commercially available flavonols tested; individual data points from radar plots shown in Figure 2; impact of F-4N to induce caspase-3 cleavage from multiple models of cellular senescence; human and mouse plasma stability of F-4N; ex vivo efficacy of F-4N in mouse PCLS; mouse microsomal stability studies with F-4N; ^1^H and ^13^C NMR characterization of all novel flavonols; and list of primers used in these studies (PDF)
