# Supplementary material for: Optimized Synthetic Flavonols Support Senescence Clearance and Lung Fibrosis Resolution
Source: ACS Pharmacol Transl Sci. 2025 Sep 4;8(9):3033–46. doi: 10.1021/acsptsci.5c00231 (PMC12441843; doi:10.1021/acsptsci.5c00231)
Supplement: Supplementary file 1 [file pt5c00231_si_001.pdf]

Supporting Information

Optimized synthetic flavonols support senescence clearance and lung fibrosis resolution

Jeffrey A. Meridew[1], John A. Vu[2,3], Daniela Chow[4], Ana Maria Diaz Espinosa[1], Namita Saraf[1], Ashley Y. Gao[1], Jair Machado Espindola-Netto[1,5], Sara Dresler[1], Madison G. Whaley[1], Kyoung M. Choi[1], Yong Li[1], Helene Martini[1,5], Eva Carmona Porquera[6], Patrick A. Link[1], Thomas M. Kollmeyer[4], Joao F. Passos[1,5], Marissa J. Schafer[1,5], Nathan K. LeBrasseur[1,5], Daniel J. Tschumperlin[1], Andrew J. Haak*[1,4,]

[1]Department of Physiology and Biomedical Engineering, Mayo Clinic, Rochester, MN 55902

[2]College of Medicine and Science, Mayo Clinic, Rochester, MN 55905

[3]Center for Clinical and Translational Science, Rochester, MN 55902

[4]Department of Molecular Pharmacology and Experimental Therapeutics, Mayo Clinic, Rochester, MN 55902

[5]Robert and Arlene Kogod Center on Aging, Mayo Clinic, Rochester, MN 55905

[6]Thoracic Diseases Research Unit, Departments of Medicine and Biochemistry, Mayo Clinic College of Medicine, Rochester, MN 55905

*Corresponding author: haak.andrew@mayo.edu

Table of Contents

3-point dose-response for the original 30 commercially available flavonols tested……………………………………..3

Individual datapoints from radar plots shown in Figure 2……………………………………………………………….4

Impact of F-4N to induce caspase-3 cleavage from multiple models of cellular senescence………………………..5

Human and mouse plasma stability of F-4N……………………………………………………………………………..6

Ex vivo efficacy of F-4N in mouse PCLS…………………………………………………………..………………..7

Mouse microsomal stability studies with F-4N……………………………………………………………….………..8

^{1}H and ^{13}C NMR characterization of all novel flavonols……………………………………………………………9-10

Primers used in these studies…………………………………………………………………………………………11

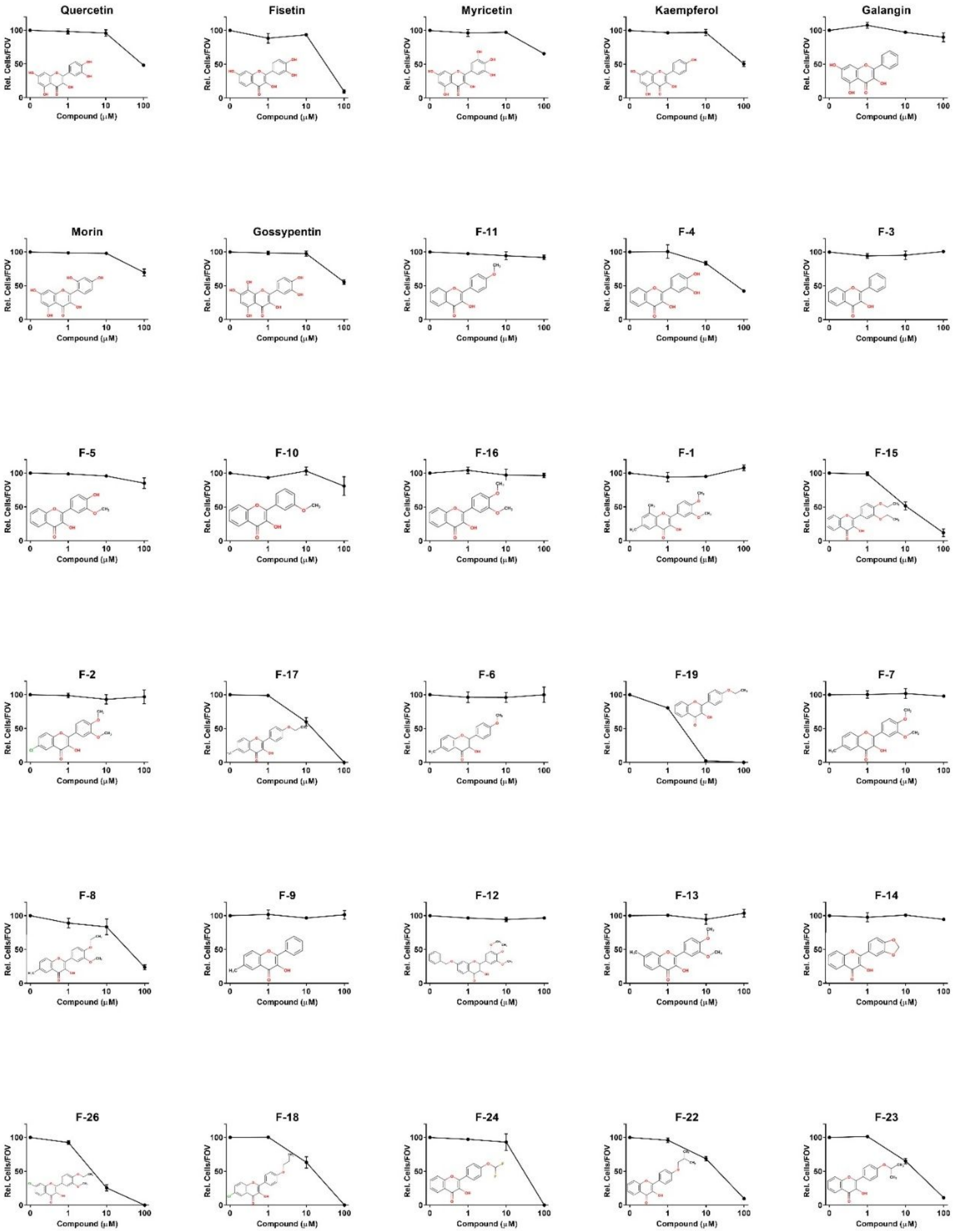

Supplemental Figure 1. Synthetic flavonols containing a para-ethoxy substitution on the B ring reduce cell number in senescent lung fibroblasts. Human lung fibroblasts were passaged in culture (P=18-20) then treated for 72 hours with the indicated flavonol (1, 10, and 100μM). After incubation cells were fixed and stained with DAPI. Cells/Field of view were calculated using automated software. N=3 biologically independent experiments.

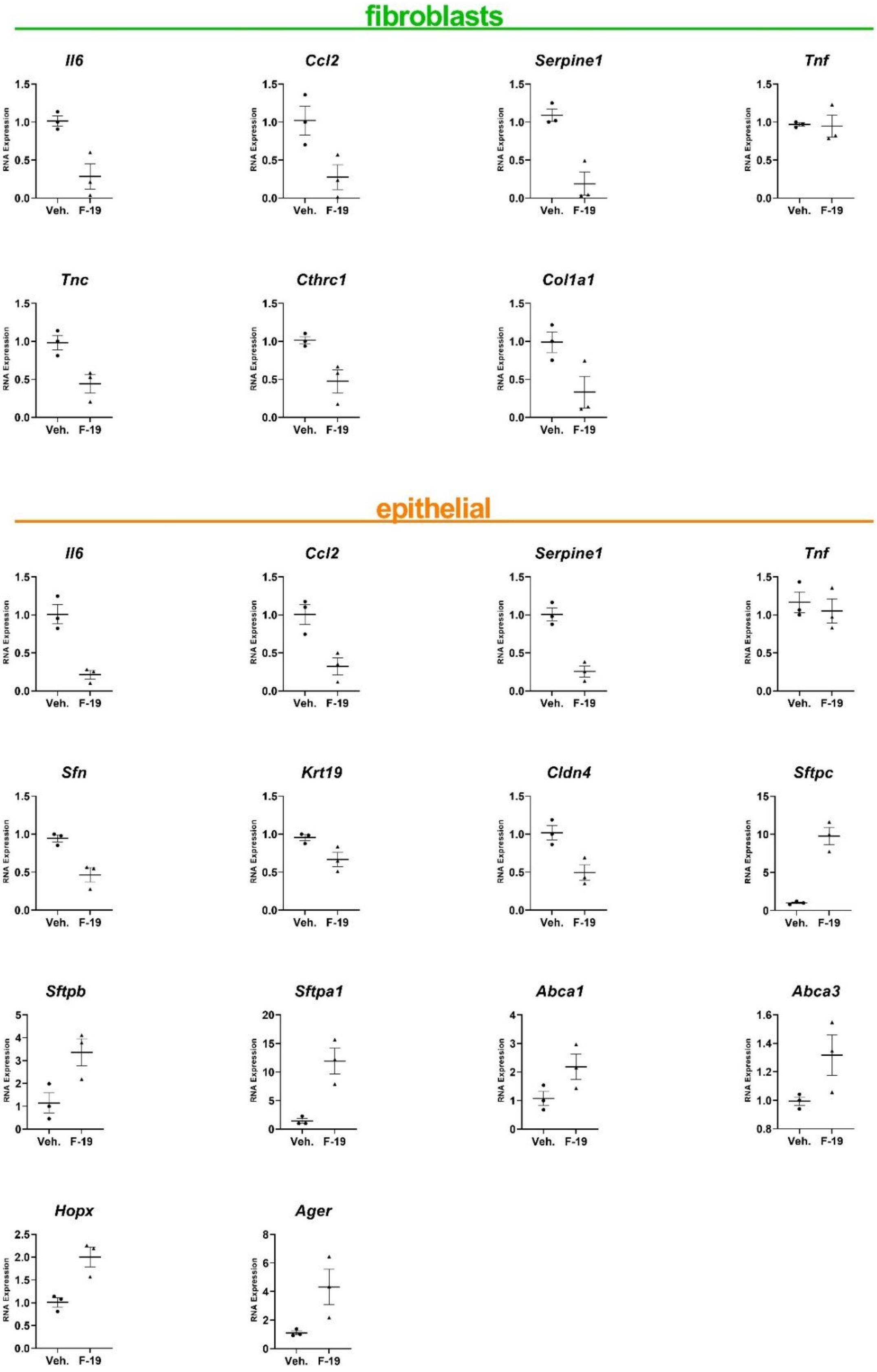

Supplemental Figure 2. Individual datapoints from radar plots shown in Figure. 2.

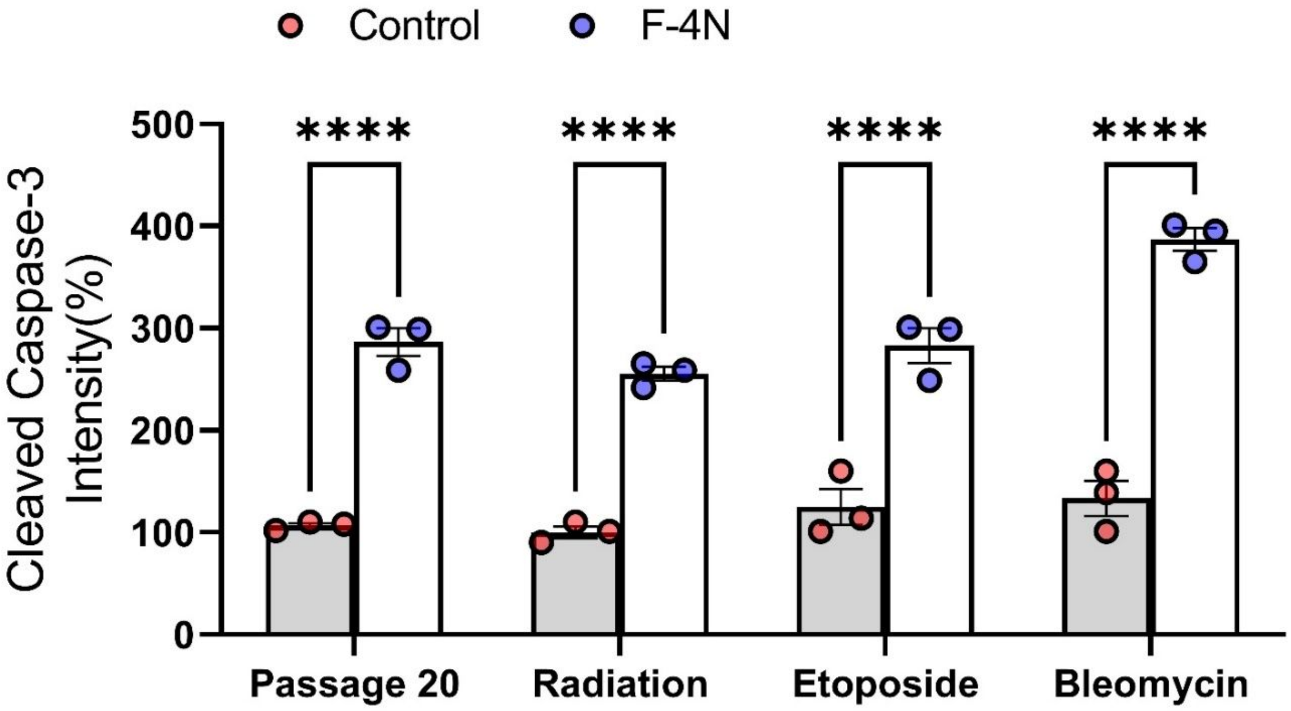

Supplemental Figure 3. F-4N induces cleaved caspase-3 expression in senescent fibroblasts derived from multiple stimuli. Senescence was induced by replication, radiation (20 Gy of X-ray), etoposide (20μM), or bleomycin (10μ). Cells were then incubated for 72 hours +/- F-4N (3μM) prior to fixation and staining for cleaved caspase-3 and DAPI. N=3 independent experiments (**** $p < 0.0001$, vs. the indicated group).

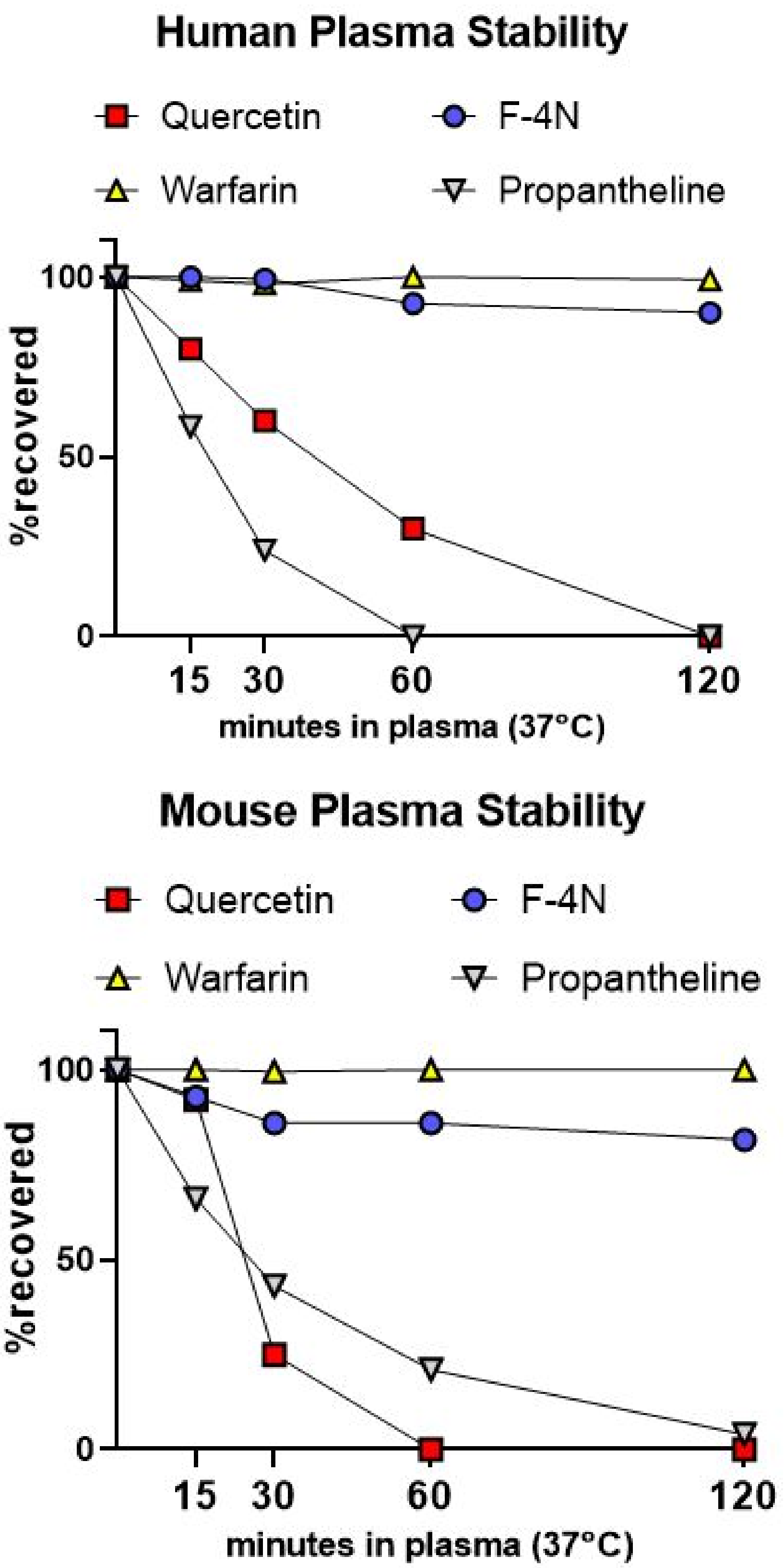

Supplemental Figure 4. Plasma stability assay. Compounds were incubated with human (top) or mouse (bottom) plasma for the indicated amount of time, %recovered was analyzed by LC-MS. Warfarin as a control for stable compound, propantheline as a control for an unstable compound. Analysis performed under contract by Cyprotex (Framingham, MA).

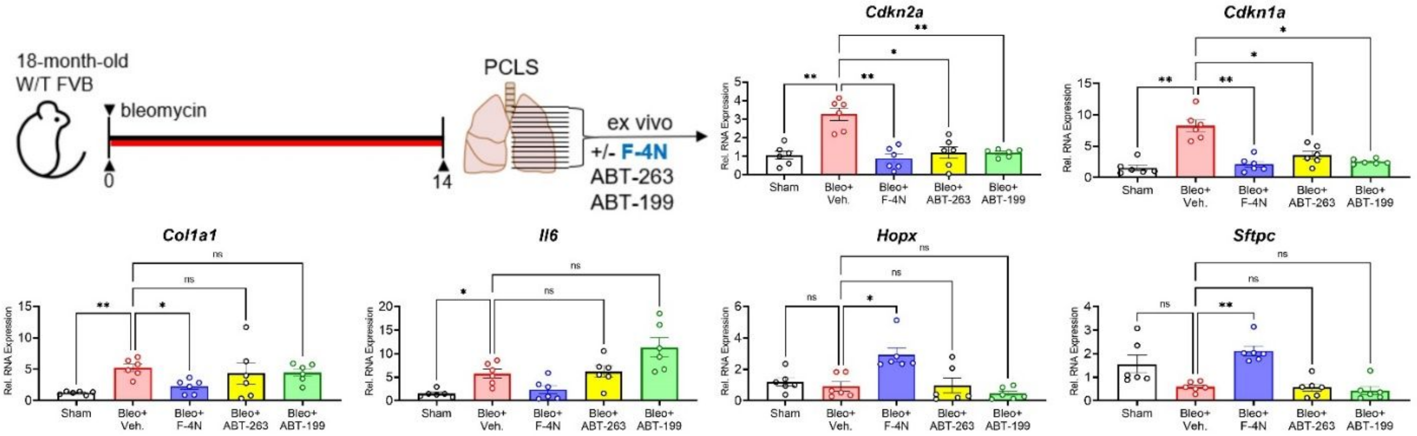

Supplemental Figure 5. Synthetic flavonol F-4N supports clearance of senescence associated markers ex vivo. Aged (18-month-old) mice were administered intratracheal “sham” or bleomycin (Bleo) and 14 days after exposure the left lobe was harvested to generate 300μm precision-cut lung slices that were cultured for 72 hours with F-4N (3μM), ABT-263 (5.0μM), or ABT-199 (3μM) ex vivo, prior to homogenization, RNA isolation and qPCR analysis. N=6 sham or bleomycin mice. Comparisons made using ANOVA, * $p < 0.05$, ** $p < 0.01$ vs. the indicated group.

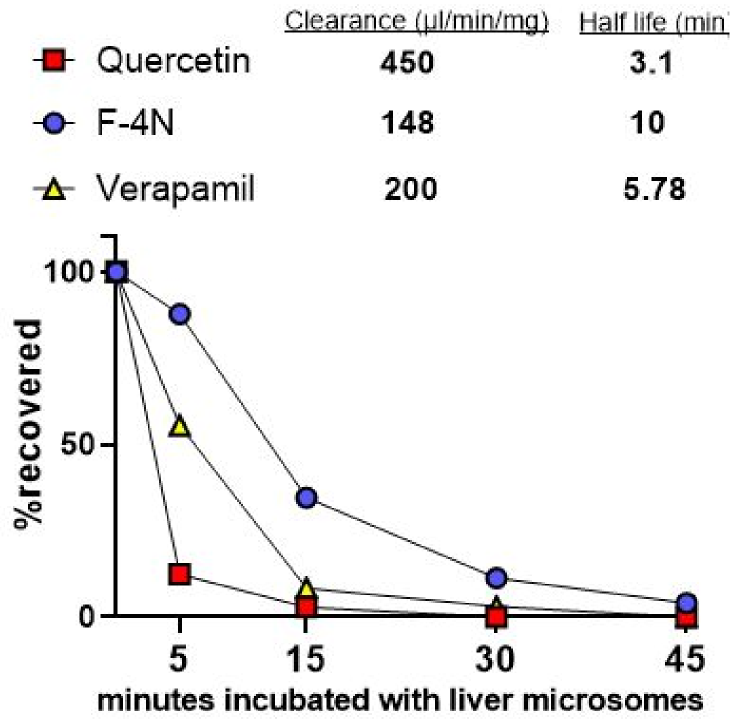

Supplemental Figure 6. Mouse in vitro microsomal stability studies. Compounds were incubated with mouse derived liver microsomes for the indicated amount of time, %recovered was analyzed by LC-MS. Verapamil is shown as a reference compound known to have a relatively short half-life in microsomes. Analysis performed under contract by Cyprotex (Framingham, MA).

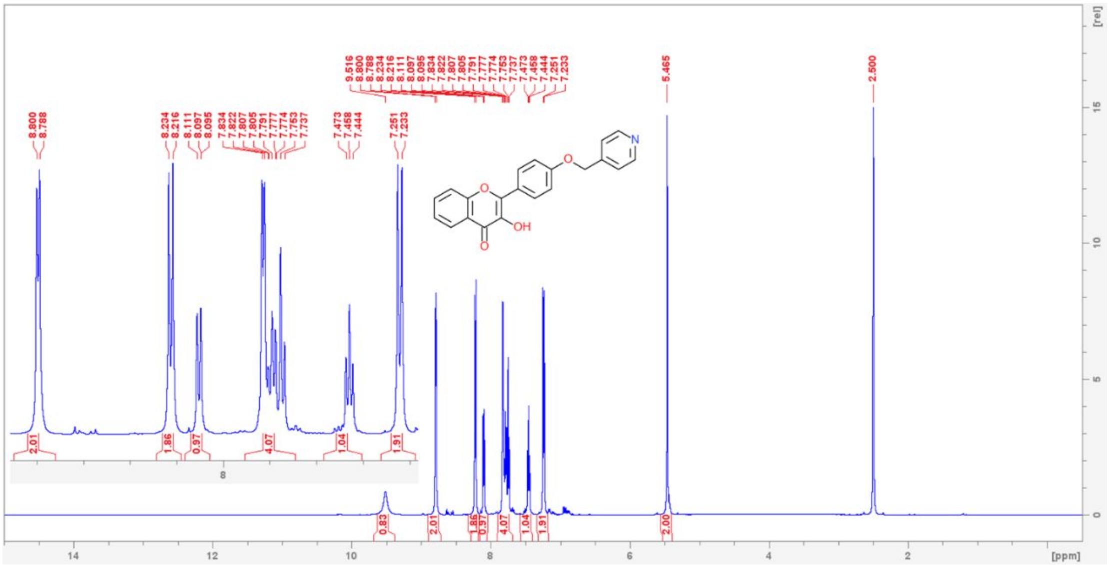

Supplemental Figure 7. ^{1}H NMR spectrum of compound F4N (500 MHz, DMSO-*d*6)

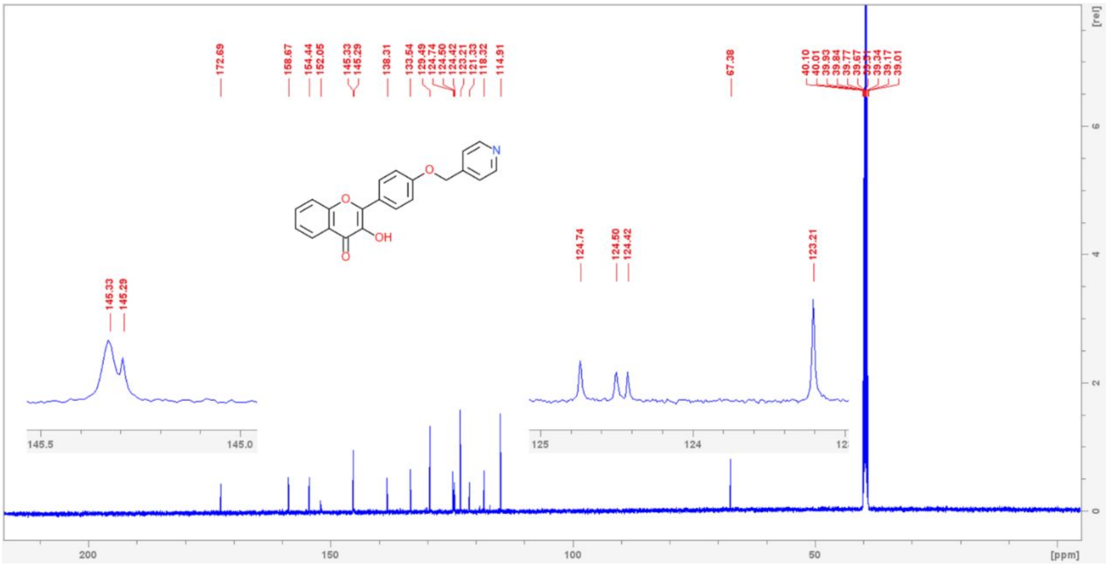

Supplemental Figure 8. ^{13}C NMR spectrum of compound F4N (126 MHz, DMSO-*d*6)

3-hydroxy-2-{4-[(pyridin-4-yl)methoxy]phenyl}-4H-chromen-4-one (**F-4N**)

^{1}H NMR (500 MHz, DMSO-d_6) δ 9.52 (bs, 1H), 8.79 (d, 2H, *J* = 5.9 Hz), 8.22 (dd, 2H, *J* = 7.8, *J* = 0.9 Hz), 8.10 (dd, 1H, *J* = 7.1, *J* = 0.9 Hz), 7.83 (d, 2H, *J* = 5.7 Hz), 7.77-7.81 (m, 1H), 7.73-7.77 (m, 1H), 7.44-7.45 (m, 1H), 7.24 (d, 2H, J = 9.0 Hz), 5.46 (s, 2H). ^{13}C{1H} NMR (126 MHz, DMSO-d_6) δ 172.7, 158.7, 154.4, 152.1, 145.32, 145.29, 138.3, 133.5, 129.4, 124.7, 124.50, 124.42, 123.2, 121.3, 118.3, 114.9, 67.8.

NMR peak descriptions for newly synthesized flavonols in Table 1

2-(4-ethoxyphenyl)-3-hydroxy-4H-chromen-4-one (**F-19**)

^{1}H NMR (600 MHz, DMSO-d_6) δ 10.24 (s, 1H), 7.94 (d, J = 2.2 Hz, 1H), 7.69 (dd, J = 8.7, 2.2 Hz, 1H), 7.52 (d, J = 8.7 Hz, 1H), 7.44 (d, J = 8.7 Hz, 2H), 6.91 (d, J = 8.7 Hz, 2H), 6.55 (s, 1H), 4.04 (q, J = 7.0 Hz, 2H), 1.38 (t, J = 7.0 Hz, 3H). ^{13}C NMR (150 MHz, DMSO-d_6) δ 178.2, 162.4, 156.5, 154.8, 151.0, 134.2, 130.3, 126.9, 124.0, 121.7, 116.4, 114.6, 104.5, 98.6, 63.6, 14.6.

3-hydroxy-2-(4-propoxyphenyl)-4H-chromen-4-one (**F-19(Me)**)

^{1}H NMR (600 MHz, DMSO-d_6) δ 10.23 (s, 1H), 7.95 (d, J = 2.2 Hz, 1H), 7.70 (dd, J = 8.7, 2.2 Hz, 1H), 7.53 (d, J = 8.7 Hz, 1H), 7.45 (d, J = 8.6 Hz, 2H), 6.94 (d, J = 8.6 Hz, 2H), 6.55 (s, 1H), 3.92 (t, J = 6.6 Hz, 2H), 1.68 (sext, J = 7.4 Hz, 2H), 0.94 (t, J = 7.4 Hz, 3H). ^{13}C NMR (150 MHz, DMSO-d_6) δ 178.2, 162.4, 156.6, 154.9, 151.1, 134.3, 130.2, 126.8, 124.0, 121.6, 116.3, 114.5, 104.5, 98.6, 69.7, 22.4, 10.5.

2-[4-(benzyloxy)phenyl]-3-hydroxy-4H-chromen-4-one (**F-20**)

^{1}H NMR (600 MHz, DMSO-d_6) δ 10.25 (s, 1H), 8.00 (d, J = 2.2 Hz, 1H), 7.74 (dd, J = 8.7, 2.2 Hz, 1H), 7.56 (d, J = 8.7 Hz, 1H), 7.47–7.36 (m, 5H), 7.12 (d, J = 8.6 Hz, 2H), 6.95 (d, J = 8.6 Hz, 2H), 6.56 (s, 1H), 5.12 (s, 2H). ^{13}C NMR (150 MHz, DMSO-d_6) δ 178.3, 162.6, 156.5, 154.7, 151.2, 137.8, 134.5, 130.4, 128.6, 128.3, 127.1, 124.1, 121.9, 116.6, 114.8, 104.4, 98.5, 70.2.

3-hydroxy-2-{4-[(pyridin-2-yl)methoxy]phenyl}-4H-chromen-4-one (**F-2N**)

^{1}H NMR (600 MHz, DMSO-d_6) δ 10.21 (s, 1H), 8.61 (ddd, J = 4.8, 1.7, 0.9 Hz, 1H), 8.08 (dd, J = 8.0, 1.2 Hz, 1H), 7.87 (td, J = 7.7, 1.7 Hz, 1H), 7.70 (d, J = 2.2 Hz, 1H), 7.62 (dd, J = 8.7, 2.2 Hz, 1H), 7.52 (d, J = 8.7 Hz, 1H), 7.45 (d, J = 8.6 Hz, 2H), 7.19 (d, J = 8.6 Hz, 2H), 6.55 (s, 1H), 5.31 (s, 2H).^{13}C NMR (150 MHz, DMSO-d_6) δ 178.2, 162.5, 156.9, 154.1, 150.2, 147.9, 146.0, 137.6, 134.2, 130.7, 126.9, 124.0, 121.8, 116.4, 114.7, 104.6, 98.6, 65.2.

3-hydroxy-2-{4-[(pyridin-3-yl)methoxy]phenyl}-4H-chromen-4-one (**F-3N**)

^{1}H NMR (600 MHz, DMSO-d_6) δ 10.22 (s, 1H), 8.74 (dd, J = 4.8, 1.2 Hz, 1H), 8.50 (d, J = 2.2 Hz, 1H), 7.82 (dd, J = 8.0, 2.2 Hz, 1H), 7.65 (d, J = 8.7 Hz, 2H), 7.54 (d, J = 8.7 Hz, 2H), 7.39 (dd, J = 8.0, 4.8 Hz, 1H), 7.28 (s, 1H), 6.55 (s, 1H), 5.29 (s, 2H). ^{13}C NMR (150 MHz, DMSO-d_6) δ 178.1, 162.4, 157.0, 153.5, 150.3, 147.8, 144.6, 134.1, 130.6, 127.0, 123.7, 121.5, 116.3, 114.6, 104.7, 98.5, 64.9.

3-hydroxy-2-{4-[(pyridin-4-yl)methoxy]phenyl}-4H-chromen-4-one (**F-4N**)

^{1}H NMR (600 MHz, DMSO-d_6) δ 10.24 (s, 1H), 8.82 (d, J = 4.9 Hz, 2H), 7.71 (d, J = 2.2 Hz, 1H), 7.65 (dd, J = 8.7, 2.2 Hz, 1H), 7.52 (d, J = 8.7 Hz, 1H), 7.46 (d, J = 8.5 Hz, 2H), 7.18 (d, J = 8.5 Hz, 2H), 6.55 (s, 1H), 5.26 (s, 2H). ^{13}C NMR (150 MHz, DMSO-d_6) δ 178.2, 162.3, 156.7, 152.8, 151.0, 147.4, 134.3, 130.5, 127.2, 123.8, 121.7, 116.5, 114.7, 104.5, 98.6, 65.4.

Mouse Gene Name	Forward	Reverse
Gusb	CACCCCTACCACTTACATCG	ACTTTGCCACCCTCATCC
Il6	CAAAGCCAGAGTCCTTCAGAG	GTCCTTAGCCACTCCTTCTG
Ccl2	GTCCCTGTCATGCTTCTGG	GCTCTCCAGCCTACTCATTG
Serpine1	TGCAAAAGGTCAGGATCGAG	ATTGTCTCTGTCGGGTTGTG
Tnf	CTTCTGTCTACTGAACTTCGGG	CAGGCTTGTCACTCGAATTTTG
Cthrc1	GAATGTTCAGGACCTCTTCCC	ACATCTACCAATCCAGCACC
Col1a1	ATCATAGCCATAGGACATCTGG	CTGGACAGCCTGGACTTC
Sfn	GTGAAAGAGTACCGGGAGAAG	GTAGTCACCCTTCATCTTCAGG
Krt19	CTCCCGAGATTACAACCACTAC	GTTCTGTCTCAAACTTGGTTCTG
Cldn4	GTTCATCGTGGCAAGCATG	CCATAGGGTTGTAGAAGTCGC
Sftpc	TTGTCGTGGTGATTGTAGGG	TGGAAAAGGTAGCGATGGTG
Sftpb	CTGCCCCTGGTTATTGACTAC	AGCAGAGGGTTTGGAACG
Sftpa1	GAACATGGAGACAAGGGAGAG	TGTTTGATCTCGTAGAGTGCAG
Abca1	TGACATGGTACATCGAAGCC	GATTTCTGACACTCCCTTCTGG
Abca3	CAAGAACTGTCGTAGGGAAGG	TCTTGTCCTTATTGCCCACTTG
Hopx	CCACGCTGTGCCTCATC	TGGCTCCCTAGTCCGTAAC
Ager	ACCCATCCTACCTTCTCCTG	GAGCGACTATTCCACCTTCAG
Cdkn2a	GTGCGATATTTGCGTTCCG	TCTGCTCTTGGGATTGGC
Cdkn1a	CTTGCACTCTGGTGTCTGAG	GCACTTCAGGGTTTTCTCTTG
Mmp7	CTCTCAGAATGTGGAGTATGCAG	CATGACCTAGAGTGTTCCCTG
Infa2	GAGAGAAGAAACACAGCCCC	AGCAAGTTGACTGAGGAAGAC
Ccl19	CGCATCATCCGAAGACTGAAG	TTTACTCAAGACACAGGGCTC
Cxcl13	AGATCGGATTCAAGTTACGCC	ACAGACTTTTGCTTTGGACATG
Tgfb2	TGCTAACTTCTGTGCTGGG	GCTTCGGGATTTATGGTGTTG
Ltb	CTGCGGATTCTACACCAGATC	TTGCCCACTCATCCAAGC
**Human Gene Name**	**Forward**	**Reverse**
CDKN2A	GATGTCGCACGGTACCTG	TCTCTGGTTCTTTCAATCGGG
CDKN1A	TGTCCGTCAGAACCCATGC	AAAGTCGAAGTTCCATCGCTC
GAPDH	GGAAGGGCTCATGACCACAG	ACAGTCTTCTGGGTGGCAGTG
